# Congenital Extremely Short Bowel with Atresia

**DOI:** 10.21699/jns.v6i2.574

**Published:** 2017-04-15

**Authors:** AK Sharma

**Affiliations:** Senior Pediatric Surgeon, Fortis Escort Hospital and Narayana Multi Specialty Hospital

A premature neonate, born at 32 weeks gestation to a primigravida, weighing 1.3 kg was admitted with bilious vomiting on 3^rd^ day of life. The mother never had an antenatal care and the local birth attendant in the village had conducted the delivery. The baby’s general condition was poor. He was diagnosed to have duodenal atresia; no other associated anomaly was apparent. He was septicemic; blood culture grew candida non-albicans. Exploration revealed grossly dilated stomach and duodenum; the duodenum abruptly ended at its third part. There was complete absence of small bowel and mesentery. There was small tubular structure of 2 cm size was attached to the caecum representing terminal ileum. Appendix and colon looked normal, and so were liver, pancreas and biliary duct system. Duodeno-colic anastomosis was done. 

Volvulus of small bowel has been known to result in jejuno-ileal atresias [1]. It seems that an early fetal volvulus has led to gangrene and resorption of midgut and its mesentery in the index case. The only theoretical treatment that one can think of is small bowel transplantation but it is not feasible at the neonatal age even in the developed world.

## Footnotes

**Source of Support:** None

**Conflict of Interest:** None

## Figures and Tables

**Figure 1:**
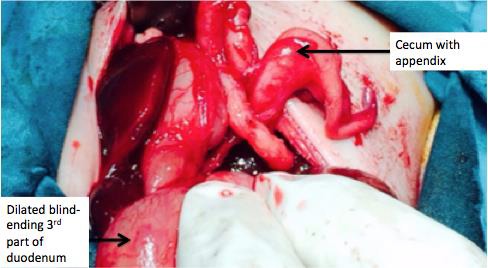
Intra-operative picture showing complete absence of the entire small bowel (from third part of duodenum to cecum) and its mesentery.
